# Effectiveness of integrative medicine practices on workers’ health within private companies

**DOI:** 10.47626/1679-4435-2020-569

**Published:** 2021-02-11

**Authors:** Koitshi Kondo, Talita Bonato de Almeida, Ronaldo Seichi Wada, Maria da Luz Rosário de Sousa

**Affiliations:** Departamento de Ciências da Saúde e Odontologia Infantil, Faculdade de Odontologia de Piracicaba, Universidade Estadual de Campinas (Unicamp) - Piracicaba (SP), Brazil

**Keywords:** health promotion, integrative medicine, occupational medicine, acupuncture therapy

## Abstract

**Introduction:**

The attention to workers’ health is fundamental not only considering economical aspects (reducing absenteeism) but also for guaranteeing their quality of life.

**Objectives:**

To verify whether integrative medicine practices are effective for workers in private companies.

**Methods:**

Data were obtained from medical records of the acupuncture service of Irmandade da Santa Casa de Misericórdia de Piracicaba, in the state of São Paulo, Brazil. This is a descriptive study that evaluated the effectiveness of invasive and non-invasive integrative medicine practices in workers’ health through the use of the visual numeric scale for measuring pain, as well as independent variables (sex, age, types of physical and emotional complaints). Our sample consisted of 259 workers in 14 companies. Results were statistically evaluated using a Student’s t-test and a significance level of p = 0.05.

**Results:**

Most of the patients that sought integrative medicine services were women (73%), with a mean age of 38 years. The mean visual numeric scale value for workers that had physical pain complaints at the first session was 4.96, while that at the final session was 1.38; this reduction was significant (p < 0.0001). Emotional symptoms showed a visual numeric scale reduction of 5.18 to 1.90 (p < 0.0001). Regardless of the practice type (invasive or non-invasive), we observed a reduction in visual numeric scale values over integrative medicine sessions, although invasive therapies resulted in a stronger reduction than non-invasive ones (p < 0.0001).

**Conclusions:**

Overall, integrative medicine practices had a positive impact on workers’ health, reducing physical and emotional pain.

## INTRODUCTION

Since the end of the 20th century, numerous actions taken by the different classes of workers have contributed to changes in public policies for workers’ health at all levels. In Brazil, in spite of the currently faced economic obstacles, the establishment of programs for the maintenance and recovery of workers’ health could contribute to improving their quality of life (QoL).^^1^^

The promotion of health and QoL in the workplace has become a part of the organizational culture of large corporations over the last few decades.^^2^^ The purpose of these programs is to amplify the possibilities of individual and collective actions on factors that interfere on workers’ health and QoL, making themselves responsible for controlling this process.^^3^^

In this context, private companies that invest in health promotion and prevention programs present different dynamics; assertive actions, when adequately planned and developed, are capable of improving the health status in all dimensions and consequently change paradigms, for example with increases in productivity and reductions in expenses with medical care.^^4^^

In the last decade, the use of integrative and complementary practices has significantly increased within the Brazilian Unified Health System (SUS) as a form of treatment and health care in a holistic and responsible manner, particularly after two ministerial administrative rulings in 2006 and 2018^^5^,^6^^ and the institution of the National Policy of Integrative and Complementary Practices (Política Nacional de Práticas Integrativas e Complementares [PNPIC]). This policy regulated these practices within the SUS - including acupuncture, homeopathy, anthroposophical medicine, and phytotherapy.^^7^^

Initiatives that promote the integrality of workers’ health have been established in private and public workplaces, benefiting from QoL programs and integrative medicine practices and focused on a broader dimension than only the worker’s health, since these worksites have an active occupational medicine service.^^8^,^9^^

Several companies and institutions worldwide aim to propose a more pleasant working environment and improve workers’ health.^^10^^ A recently published systematic review highlighted the use of several interventions such as yoga, music therapy, and mindfulness in the management of occupational pains and in the cognitive recovery of employees in companies of different segments.^^11^^

An analysis of the literature verified that studies have evaluated the use of integrative medicine practices in the public service,^^12^,^13^^ but not in private companies. Considering that integrative medicine practices may contribute to maintaining and recovering the physical and emotional wellbeing of workers and in view of an absence of studies on integrative practices for relieving workers’ complaints, the aim of the present study was to evaluate the effectiveness of integrative medicine practices on the health of workers in private companies.

## METHODS

The study was conducted with secondary data collected from specific medical records of the Health Prevention and Promotion Center (Centro de Prevenção e Promoção da Saúde), named Smart Health (Saúde Inteligente), at Irmandade da Santa Casa de Misericórdia de Piracicaba, state of São Paulo, Brazil. Data were obtained in accordance with the rules and ethical guidelines of resolution No. 196/1996 of the National Health Council of the Brazilian Ministry of Health, and were submitted to the research ethics committee (CEP/CAAE: 71628117.5.0000.5418) of the Dentistry school of Piracicaba (FOP/Unicamp).

The data used in this study was provided by medical records of 17 companies insured by the Saúde Inteligente program, which were filed within the Health Committee for Companies (Comitê de Saúde nas Empresas) of Irmandade da Santa Casa de Misericórdia de Piracicaba.

The researched population consisted of workers at the 17 registered companies,^^14^^ which comprised the commerce, manufacturing industry, and education sectors. Workers were invited to participate in this project according to convenience criteria and those that showed interest in participating were initially included in the study.

Our inclusion criteria were: workers should be over 18 years old, with more than four acupuncture sessions on their medical records between 2015 and 2016. The exclusion criteria were: less than four sessions and medical records with incomplete or outdated (before 2015) information.

Workers’ complaints were evaluated by a professional acupuncturist who intervened in cases that fell within her competence, initiating treatment with a traditional Chinese medicine approach. Three health care teams consisted of an acupuncturist and a nursing technician, and each company was assisted by one of the teams, once a week, for a period of 4 hours, with scheduled appointments for approximately 15 to 20 workers. Sessions lasted approximately 30 minutes, according to the complexity of the interventions.

The analyzed variables were classified as dependent and independent. The dependent variable that was used to evaluate pain intensity was the visual numeric scale (VNS).^^15^^ With the use of this scale, the participant was asked the following verbal question: “From 0 to 10, where 0 is related to the absence and 10 to the worst possible pain, how would you classify your complaint?” The VNS was applied at the beginning of the first, second, third, and last sessions.

The independent variables were: age (numerical scale); sex (male or female); pathology, which was classified into physical (non-specific back pain, low back pain, lumbar radiculopathy [sciatica], neck tension, and cervical pain) and emotional symptoms (anxiety, stress) according to the worker’s complaint; and type of integrative medicine practice (invasive or non-invasive). Data related to physical and emotional symptoms were collected through self-reports of patients who sought the service.

Invasive integrative medicine practices include procedures that rupture the natural barriers of the body or penetrate into its cavities^^16^^; in this study, invasive practices comprised systemic acupuncture (acupuncture needles sized 30 mm × 0.25 mm by Dong Bang, Dong Bang Acupuncture Inc., Chungnam, Korea) and bleeding (sterile lancets by Sterilance Medical Inc., Suzhou, China).

Non-invasive integrative medicine practices were those that did not rupture the natural barriers of the body and comprised auricular therapy with vaccaria seeds,^^17^^ cupping therapy (Dong Bang, Dong Bang Acupuncture Inc.),^^18^^ and trigger point myotherapy.^^19^^ Ethylene oxide was used to sterilize the acrylic cups when there was contact with blood, and 70% alcohol was used when there was no such contact.

Data were evaluated using BioEstat version 5.3 statistics software (Mamirauá Institute for Sustainable Development, Tefé, state of Amazonas, Brazil). The reduction in general VNS values according to sex, complaint, and technique was analyzed through a paired Student’s t-test, while the mean difference in VNS values before and after the therapies was evaluated through a t-test for independent samples. The level of significance was p = 0.05.

## RESULTS

**Figure 1:**
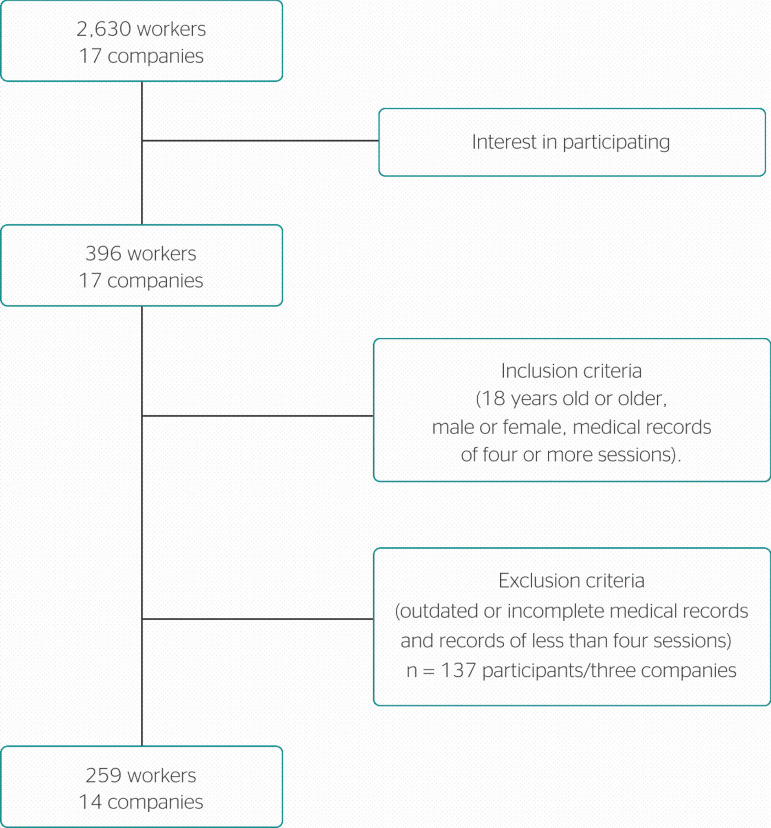
Definition of the sample and inclusion/exclusion criteria.

Our initial sample universe comprised the total number of employees in the 17 companies (n = 2630). Out of these employees, 396 sought the integrative medicine practice service. After applying the inclusion and exclusion criteria, our sample included 259 participants from 14 companies in the commerce, manufacturing industry, and education sectors (Figure 1).

The participants were between 18 and 69 years of age and mostly female (73%, as opposed to 27% male participants). The mean number of complaints reported in the first session was 2.65 and the initial mean VNS value was 5.03.

The initial mean VNS value among all patients was 5.03 and the final mean VNS was 1.56, displaying a significant reduction (p < 0.0001) in complaints of both physical and emotional pain.

Both female and male workers presented a statistically significant and similar (p < 0.069) reduction in mean VNS values between the beginning and end of treatment (female: from 4.94 to 1.59; male: from 5.24 to 1.45), indicating that both sexes benefited equally from the practices.

The complaints reported by workers were divided into two groups: physical pain and emotional complaints. The group that presented physical pain was further divided according to body areas, as seen in Table 2. The group with emotional complaints was also subdivided according to symptom occurrence (Table 2). Out of the 259 visits performed during the period considered by our study, five patients presented complaints that could not be classified as neither physical nor emotional (alcoholism and allergic rhinitis) and were therefore excluded from the statistical analysis.

**Table 1:** Number of employees (total and per company) included in the study characterized by sex, visual numeric scale (VNS) values, and number of complaints, Piracicaba, state of São Paulo, 2015-2016.

Company sector	n	Sex female n (%)	Sex male n (%)	Age - mean (range)	Initial VNS -mean (range)	Number of complaints - mean (range)
Industry	19	17 (89.0)	2 (11.0)	32.63 (21-58)	4.26 (2-10)	1.89 (1-3)
Industry	27	25 (92.5)	2 (7.5)	34.88 (19-57)	4.62 (2-9)	1.92 (1-4)
Industry	18	11 (61.1)	9 (38.9)	35.38 (21-44)	5.44 (4-6)	2.44 (1-4)
Industry	36	28 (78.0)	8 (22.0)	45.69 (24-69)	6.00 (2-9)	1.97 (1-4)
Industry	10	1 (10.0)	9 (90.0)	30.30 (24-52)	5.60 (2-7)	1.80 (1-2)
Commerce	17	17 (100.0)	0 (0)	35.41 (18-59)	5.52 (4-6)	2.35 (2-4)
Commerce	12	11 (92.0)	1 (8.0)	33.50 (24-56)	3.08 (1-6)	2.30 (1-3)
Commerce	15	7 (47.0)	8 (53.0)	41.46 (25-58)	5.80 (5-7)	2.66 (1-4)
Commerce	11	3 (27.3)	8 (72.7)	40.00 (25-53)	3.63 (3-6)	3.09 (1-5)
Commerce	34	27 (79.5)	7 (20.5)	37.17 (20-58)	5.32 (2-8)	3.41 (1-8)
Commerce	15	14 (93.0)	1 (7.0)	41.80 (21-56)	4.46 (2-9)	2.60 (1-4)
Commerce	18	13 (72.0)	5 (28.0)	37.61 (21-65)	5.88 (5-7)	2.27 (1-4)
Commerce	9	8 (89.0)	1 (11.0)	36.22 (29-52)	3.00 (2-4)	2.55 (2-4)
Education	18	9 (50.0)	9 (50.0)	44.33 (29-66)	4.83 (3-9)	2.11 (1-4)
Total	259	189 (73.0)	70 (27.0)	38.24 (18-69)	5.03 (1-10)	2.65 (1-8)

**Table 2:** Complaints reported by workers, divided by sex, Piracicaba, state of São Paulo, 2015-2016.

Type of pain/complaint		Sex n (%)	
	n (%)	Female	Male
Physical			
Neck tension, cervical pain, brachialgia	69 (38.12)	54 (78.3)	15 (21.7)
Low back pain, lumbar radiculopathy (sciatica), non-specific back pain	59 (32.6)	41 (69.5)	18 (30.5)
Pain in general*	32 (17.7)	23 (71.9)	11 (28.1)
Other^†^	21 (11.6)	16 (76.2)	8 (23.8)
Total	181 (100)	135 (74.7)	46 (25.3)
Emotional			
Anxiety	43 (58.9)	33 (77.0)	10 (23.0)
Stress	23 (31.5)	14 (61.0)	9 (39.0)
Other^‡^	7 (9.6)	5 (71.5)	2 (28.5)
Total	73 (100)	52 (71.2)	21 (28.8)

* Knee, elbow, shoulder, hip, ankle, and muscle pain.

† Headache, migraine, sinusitis, fatigue, and gastritis.

‡ Insomnia, agitation, irritability, and panic disorder.

An analysis of the mean VNS values obtained in the first (initial mean = 4.96) and last sessions (final mean = 1.38) of workers that complained of physical pain (n = 181) indicated a significant reduction in the reported symptoms (p < 0.0001). A significant reduction (from 5.18 to 1.90, p < 0.0001) was also verified among workers that complained of emotional symptoms (n = 73), highlighting the effectiveness of the employed techniques (Table 3). The reduction observed in the differences between initial and final mean VNS values were similar when comparing both groups (physical and emotional symptoms).

**Table 3:** Mean visual numeric scale (VNS) values throughout integrative medicine sessions and according to each type of complaint, Piracicaba, state of São Paulo, 2015-2016.

Type of pain/complaint	Mean values				p-value*
	1st session	2nd session	3rd session	Last session	< 0.05
Physical	4.96	3.56	2.71	1.38	< 0.0001
Emotional	5.18	3.94	3.15	1.90	< 0.0001
Total	5.03	3.69	2.86	1.56	< 0.0001

* Student's t-test with differences between the first and last sessions.

Table 4 shows the reduction in mean VNS values throughout the sessions according to sex and type of complaint reported by the worker. There was no statistical difference in total complaints between females and males (p < 0.069), thus demonstrating that integrative practices were effective for both sexes.

**Table 4:** Reduction in visual numeric scale (VNS) values throughout integrative medicine sessions and according to sex and type of complaint, Piracicaba, state of São Paulo, 2015-2016.

Complaint	Mean VNS			
	1st session	2nd session	3rd session	Last session
Female				
Physical pain	4.85	3.48	2.69	1.43
Emotional changes	5.21	4.21	3.34	2.04
Total	4.94	3.68	2.87	1.59a
				
Male				
Physical pain	5.31	3.93	2.90	1.39
Emotional changes	5.09	3.34	2.70	1.57
Total	5.24	3.75	2.84	1.45a

Student's t-test for paired samples. Similar letters in the column mean similar results.

**Figure 2:**
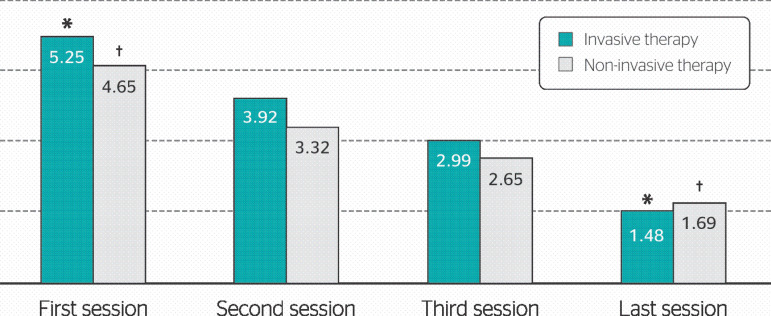
Mean visual numerical scale (VNS) values according to the type of employed integrative medicine technique. Statistical analyses used a paired Student's t-test for comparing the first and last sessions. * Pain reduction with invasive therapy (p ≤ 0.05). † Pain reduction with non-invasive therapy (p ≤ 0.05).

The mean VNS values reported in each session according to the type of employed technique are demonstrated in Figure 2.

**.**

Patients who underwent only invasive therapies (n = 161) presented a statistically significant reduction (p < 0.0001) from 5.25 to 1.48 in mean VNS values, as well as those who underwent only non-invasive integrative therapies (n = 98), who presented a reduction from 4.65 to 1.69 in mean VNS values (p < 0.0001).

The analysis of mean differences between initial and final VNS values, performed by means of a Student’s t-test for independent samples comparing the two types of therapies, demonstrated a higher reduction in pain symptoms with the use of invasive therapies in comparison with the non-invasive ones (p < 0.0002).

Out of 259 patients, 28 (9.84%) reported that the pain felt after all the integrative medicine sessions was reduced to a VNS value of 0. Among these patients, 25 had presented physical pain complaints and three had reported emotional symptoms. The remaining patients (226) reported a significant reduction in pain after the sessions, but did not report the VNS value to have reached 0; of these, 149 (66.5%) still had complaints of physical pain and 77 (33.5%), of some type of emotional change.

## DISCUSSION

The integrative medicine therapies evaluated by this study were effective in reducing patient complaints; this was true for both invasive (systemic acupuncture and bleeding) and non-invasive therapies (auriculotherapy, cupping therapy, and myotherapy).

Significantly increasing rates of work-related diseases are reported within worker classes each year, and there is not enough available data for a detailed study of the topic regarding the Brazilian situation. Nevertheless, prevalence studies have allowed us to make an approximate estimate, and according to data from the Brazilian Social Security Institute, most of the sickness benefits issued in recent years have been attributed to musculoskeletal disorders. Assumpção & Abreu^^20^^ reported that 50% of the evaluated bank workers in Pelotas, state of Rio Grande do Sul (RS), Brazil, complained of musculoskeletal pain, while 75% of the studied metalworkers in Canoas (RS) complained of some type of musculoskeletal pain.

A pioneer American research regarding the use of complementary and alternative medicine services for the treatment of migraine and headaches revealed that women accessed these services more frequently than men.^^21^,^22^^ Nevertheless, our results indicated that the VNS values were similar between men and women (p < 0.069), demonstrating that integrative medicine practices were effective in both sexes.

Most of the workers that sought care by the alternative medicine service had complaints of physical origins. A study conducted in an university hospital in the state of Santa Catarina, Brazil, indicated that 65% of the demand for their acupuncture service was due to complaints of musculoskeletal and connective tissue disorders,^^23^^ corroborating our findings of 69.88% complaints of musculoskeletal origin.

According to Alves et al.,^^24^^ a reduction of 80% in pain intensity occurred in patients with cervical pain after four sessions of systemic acupuncture. In the present study, patients with cervical pain reported a reduction in VNS values from 4.96 to 1.38 (p < 0.0001), which was similar to the results obtained for emotional complaints (from 5.18 to 1.90; p < 0.0001).

The mechanisms of action of invasive and non-invasive therapies differ in a few ways. In both types of therapies, there is an energetic stimulation of specific points, a mobilization of qi in acupuncture meridians, and the stimulation of efferent nerves of the central nervous system. On the other hand, in invasive therapies (such as systemic acupuncture with needles), stimulation is also performed through local cell damage and consequent release of hormones and endorphins, which desensitize the nerve fibers leading to the central nervous system and cause a reduction in symptoms. In non-invasive therapies, there is no cell injury because the epithelial barrier is not disrupted.^^25^^

In auriculotherapy using seeds, the stimulation of specific points promotes a mobilization of stagnant qi and the energy of the acupuncture meridians passes through the region, additionally producing a reflex arc effect between the auricular nerves and the corresponding organ (through the central nervous system), ultimately achieving treatment from a distance.^^17^^ Due to the particularities of its mechanism of action, systemic acupuncture with needles causes greater mobilization and energy balance than auriculotherapy with seeds, leading to a greater reduction in the symptoms reported by the patient, as demonstrated by our results.

In a comparative study including various invasive techniques and a non-invasive treatment control, randomized for treatment of chronic low back pain, patients were divided into four groups: one underwent physical therapy and the other three were subjected to electro-acupuncture, Yamamoto new scalp acupuncture (YNSA), and auricular acupuncture with semi-permanent needles.^^26^^ In addition to the oriental techniques, physical therapy was also included in the three groups, aiming to propose an integrative medicine practice. The intervention consisting of only the physical therapy practice did not have a significant effect - neither for pain control or in the degree of functional independence. The other interventions resulted in significant improvements in pain and functional independence. The most significant results were obtained with auricular therapy, with the greatest improvement in functional independence among all groups.^^26^^

Both invasive and non-invasive therapies significantly reduced the VNS values for complaints of physical pain and emotional symptoms. This was corroborated by a comparative study using systemic and auricular acupuncture in the control of presurgical anxiety before surgical extraction of the mandibular third molar: both therapies significantly reduced the patient’s state of anxiety.^^27^^ In our study, invasive interventions generated a higher reduction in symptoms when compared to non-invasive therapies.

Among the limitations of this study, we can cite the use of the VNS scale; it is a subjective measure, and the data were collected only at the beginning of each session. Furthermore, the samples of workers from each company may not represent the universe in which they were included and, as is the case for other studies that use secondary data, this is not a controlled study.

The evaluation performed in this study allowed us to describe the effectiveness of a program of integrative medicine practices in private companies with the aim of improving workers’ health. Although the Brazilian SUS is a national public health program and regulates the availability of these practices for the general population,^^5^^ there is still a need to create specific legislation for integrative medicine practices to be incorporated to health care services of private companies.

## CONCLUSION

This study demonstrated that integrative medicine practices (both invasive and non-invasive) had a positive effect on the workers’ health, reducing both physical and emotional pain.

## References

[1] Lacaz FAC (2017). Saúde dos trabalhadores: cenários e desafios. Cad Saúde Pública [Internet].

[2] Silva AQ (2017). Diagnóstico, política e programa de qualidade de vida no trabalho em uma instituição pública brasileira: a percepção dos trabalhadores como premissa para mudanças no contexto organizacional.

[3] Malta DC, Silva MMA, Albuquerque GM, Lima CM, Cavalcante T, Jaime PC (2014). A implementação das prioridades da Política Nacional de Promoção da Saúde, um balanço, 2006 a 2014. Ciênc Saúde Colet.

[4] Baicker K, Cutler D, Song Z (2010). Workplace wellness programs can generate savings. Health Aff (Millwood).

[5] Brasil, Ministério da Saúde (2006). Portaria Nº 971, de 3 de maio de 2006. Aprova a Política Nacional de Práticas Integrativas e Complementares (PNPIC) no Sistema Único de Saúde.

[6] Brasil, Ministério da Saúde (2006). Portaria Nº 1.600, de 17 de julho de 2006. Aprova a constituição do Observatório das Experiências de Medicina Antroposófica no Sistema Único de Saúde (SUS).

[7] Brasil, Ministério da Saúde, Secretaria de Atenção à Saúde, Departamento de Atenção Básica (2015). Política Nacional de Práticas Integrativas e Complementares no SUS: ATITUDE DE AMPLIAÇÃO DE ACESSO.

[8] Silva LB, Lima IC, Bastos RA (2016). Terapias Complementares e Integrativas: Conhecimento e utilização pelos docentes do curso de enfermagem de uma Instituição Pública. Rev saúde col UEFS.

[9] Iorio RC, Alvarenga AT, Yamamura Y (2004). Acupuntura no Currículo Médico: Visão de Estudantes de Graduação em Medicina. Rev Bras Educ Med.

[10] Ahlstrom L, Hagberg M, Dellve L (2013). Workplace Rehabilitation and Supportive Conditions at Work: A Prospective Study. J Occup Rehabil.

[11] Verbeek J, Ruotsalainen J, Laitinen J, Korkiakangas E, Lusa S, Mänttäri S (2019). Interventions to enhance recovery in healthy workers; a scoping review. Occup Med (Lond).

[12] Cintra MER, Figueiredo R (2010). Acupuntura e promoção de saúde: possibilidades no serviço público de saúde. Interface - Comunic, Saude, Educ.

[13] Vasconcellos P (2019). Acupuntura como forma de tratamento no sistema único de saúde. FJH.

[14] Instituto Brasileiro de Geografia e Estatística - IBGE (2017). Cadastro Central de Empresas.

[15] Ciena AP, Loth EA, Picanço VV, Pacini VC, Gatto R, Magno IMN (2008). Influência da intensidade da dor sobre as respostas nas escalas unidimensionais de mensuração da dor em uma população de idosos e de adultos jovens. Semina: Ciênc Biol Saúde.

[16] Sousa CMM, Moura MEB, Santos AMR, Nunes BMV, Alves MSCF (2009). Responsabilidade civil dos profissionais de enfermagem nos procedimentos invasivos. Rev Bras Enferm.

[17] Gonzales EG (2003). Auriculoterapia - Escola Huang Li Chun.

[18] Chira IZ (2001). Ventosaterapia, medicina tradicional chinesa.

[19] Nascimento FR, Cunha SG, Cardoso SR (2013). A aplicação da inibição muscular com a mioterapia para o alivio da dor e a promoção da qualidade de vida em um ambiente ocupacional. Rev Inspirar Movim Saude.

[20] Assunção AA, Abreu MNS (2017). Factor associated with self-reported work-related musculoskeletal disorders in Brazilian adults. Rev Saude Publica.

[21] Rhee TG, Harris IM (2017). Gender Differences in the Use of Complementary and Alternative Medicine and Their Association With Moderate Mental Distress in US Adults With Migraines/Severe Headaches. Headache.

[22] Kristoffersen AE, Stub T, Salamonsen A, Musial F, Hamberg K (2014). Gender differences in prevalence and associations for use of CAM in a large population study. BMC Complement Altern Med.

[23] Lima JHC (2007). Estudo da demanda de tratamento por acupuntura no Hospital Universitário da Universidade Federal de Santa Catarina.

[24] Alves AKCR, Silva RAF, Licurci MGB, Fagundes AA (2013). Efeito da acupuntura sistêmica na intensidade da dor de pacientes com cervicalgia. Rev Univap.

[25] Lent R (2010). Cem bilhões de neurônios: conceitos fundamentais de neurociências.

[26] Mehret MOC, Colombo CCG, Silvério-Lopes S (2010). Estudo comparativo entre as técnicas de acupuntura auricular, craneoacupuntura de Yamamoto, eletroacupintura e cinesioterapia no tratamento da lombalgia crônica. Rev Bras Terap Saúde.

[27] Fonseca LM (2009). Avaliação comparativa da acupuntura sistêmica e auricular no controle da ansiedade pré-operatória em cirurgias odontológicas de 3º molar inferior.

